# Stability of Auditory Discrimination and Novelty Processing in Physiological Aging

**DOI:** 10.3233/BEN-120261

**Published:** 2013

**Authors:** Alberto Raggi, Domenica Tasca, Francesco Rundo, Raffaele Ferri

**Affiliations:** Department of Neurology I.C.Oasi Institute for Research on Mental Retardation and Brain Aging (IRCCS)TroinaItaly

**Keywords:** Normal aging, event-related potentials, ERPs, auditory discrimination, novelty processing

## Abstract

Complex higher-order cognitive functions and their possible changes with aging are mandatory objectives of cognitive neuroscience. Event-related potentials (ERPs) allow investigators to probe the earliest stages of information processing. N100, Mismatch negativity (MMN) and P3a are auditory ERP components that reflect automatic sensory discrimination. The aim of the present study was to determine if N100, MMN and P3a parameters are stable in healthy aged subjects, compared to those of normal young adults. Normal young adults and older participants were assessed using standardized cognitive functional instruments and their ERPs were obtained with an auditory stimulation at two different interstimulus intervals, during a passive paradigm. All individuals were within the normal range on cognitive tests. No significant differences were found for any ERP parameters obtained from the two age groups. This study shows that aging is characterized by a stability of the auditory discrimination and novelty processing. This is important for the arrangement of normative for the detection of subtle preclinical changes due to abnormal brain aging.

## 1. Introduction

The progressive and rapid aging of population is a relevant demographic feature in the Western countries and age-related diseases are becoming an increasingly important clinical issue. Therefore, in the neurosciences, the effects of aging on cognitive changes are nowadays mandatory research targets; moreover, normative data for clusters of the population and potential diagnostic biomarkers of preclinical and mild cognitive decline in pre-senile subjects are needed in order to establish early pharmacological and behavioral treatments.

Psychophysiological methods, such as event-related potentials (ERPs) may represent a tool useful to assess age-related changes in information processing speed and capability [1,2]. ERPs are voltage fluctuations that are associated in time with some mental or physical occurrence. These potentials can be recorded from the human scalp and extracted from the ongoing electroencephalogram by means of filtering and signal averaging. ERPs allow the study of the physiological source of psychological processes during their dynamics [3].

The age-related features of auditory discrimination and novelty processing were the topics of this study and the ERP components considered were the N100, the Mismatch Negativity (MMN) and the P3a obtained with a passive paradigm. The N100, an early fronto-central negativity following stimulus delivery, has latency around 50–150 ms and is elicited from both rare and frequent stimuli. The effect of selective auditory attention on this component is not due solely to an enlargement of the exogenous N100 component of the vertex potential, but rather includes the addition of a prolonged endogenous component [4]. It is believed that physiological aging does not affect this attention-modulated component [5] which, for instance, is reduced in amplitude in amyotrophic lateral sclerosis [6] that is characterized by a sub-clinical frontal patholog-

ISSN 0953-4180/13/$27.50 © 2013 – IOS Press and the authors. All rights reserved ical involvement, beyond the motor areas [7,8]. The MMN is an ERP component elicited, most often in the auditory oddball paradigm, by low probability deviant stimuli embedded in a sequence of high probability standard stimuli. Since MMN is more evident when subjects ignore the auditory stimuli or perform a distracting task, it is supposed to be an index of preattentive processing. MMN is automatically generated whenever there is a mismatch between the neuronal model of the physical features of the standard stimulus and the deviant stimulus [9,10]. MMN is ideal to address if working memory impairment is due to premature trace decay; in fact, by varying the interstimulus interval (ISI) between tones, MMN parameters can provide an index of auditory memory trace decay over time [9,10]. Decrease echoic discrimination has been detected in aging, Alzheimer's disease, Parkinson's disease, schizophrenia and aging (see [10–12] for review). In order to obtain the P3a evoked potential, novel stimuli are presented infrequently in a background of frequently occurring standard stimuli and infrequently occurring distractor stimuli, while the subject is not required to respond mentally or physically to any stimulus. The distractor tone (novelty) elicits a P3a that has a frontal/central maximum amplitude distribution and frontal sources [13,14]. Normal aging especially disrupts frontal functioning [15]; there are also some diseases, such as sleep disorders, causing mild frontal deficits that can be studied with the P3a [16,17].

The aim of this study was to analyze auditory ERP features in young adults and in aging, in the period preceding the advanced old age (pre-old) by means of the N100, the MMN and the P3a obtained with an auditory stimulation at a relatively short ISI and at a relatively long ISI. The central working hypothesis of this study was to provide data to be used in further research for comparison with groups of patients with sub- or pre-clinical cognitive deficits, with Parkinson's disease or mild cognitive impairment as examples.

## 2. Methods

### 2.1. Subjects

Fourteen (8 females) young adults and 13 (7 females) pre-old subjects were admitted to this study (see Table 1). Applicants with a history of any neurological diseases, psychiatric disorders, head trauma, alcohol or psychotropic drug use, hearing disturbance, cases of dementia in their families were excluded. All the most common diseases of elderly and their more frequent risk factors were excluded with an appropriate anamnesis.

All volunteers belonged to the local community and signed an informed consent according to the Declaration of Helsinki before entering the study which was approved by the local ethics committee.

### 2.2. Neuropsychological assessment

Handedness was measured with the Edinburgh Handedness Inventory [18]. Mood was assessed with the Hamilton Depression Scale (HDRS) [19]. The cognitive status was evaluated by means of the Mini-mental State Examination (MMSE) [20] and of the Raven's Advances Progressive Matrices [21].

### 2.3. Event-related potentials (ERPs)

Unipolar recordings of auditory evoked potentials, with reference to the linked mastoids, were obtained from electrodes at 5 sites (Fz, Cz, Pz, F3, F4), according with the 10–20 electrode system of the International Federation of Clinical Neurophysiology [22]. A ground electrode was positioned at FPz. Electrode impedances were less than 5 k$\Omega$. In order to reduce the effect of ocular movement artifacts, both horizontal and vertical ocular movements were also recorded with electrodes placed above and below the right eye and on both the outer canthi, and served for the rejection of epochs containing eye movement potentials higher than 100 $\mu$V. Each subject was presented with four sequences of stimuli with an intensity of 90 dB. Each sequence consisted of 600 tone stimuli; two were with an ISI of 800 ms and two with an ISI of 2,400 ms. Three types of stimuli were presented through headphones: (1) *standard* stimuli were 1,000 Hz sinusoidal tones of 100 ms duration and represented 75% of stimuli in each sequence; (2) deviant stimuli were 1,300 Hz sinusoidal tones of 100 ms duration and represented 15% of stimuli in each sequence, randomly presented among the standard stimuli; and (3) *novel* stimuli were complex and non-monotonal, as compared to standard and deviant stimuli, with the same duration but with a new different spectral content in each novel presentation. Also novel stimuli were presented randomly in the sequence of standard tones and represented 10% of stimuli.

The acquisition epoch length was 1,000 ms and included 200 ms before the stimulus for the baseline automatic correction. Signals were band-pass filtered at

Table 1
Demographic and neuropsychological features of subjects

| | Young adults ($n = 14$) | | Pre-old subjects ($n = 13$) | | | |
|---|---|---|---|---|---|---|
| | Mean | S.D | Mean | S.D. | ANCOVA (F) | Cohen's "d" |
| Age, years | 33.6 | 3.10 | 63.9 | 2.63 | **−27.246*** | **−10.529** |
| Education, years | 15.7 | 2.79 | 5.7 | 1.32 | **11.797*** | **4.601** |
| Mini-mental state examination | 29.1 | 1.75 | 26.4 | 1.89 | 1.309 | **1.513** |
| Edinburgh handedness inventory | 34.0 | 3.19 | 34.8 | 2.61 | 3.471 | −0.290 |
| Hamilton depression scale | 3.4 | 4.24 | 3.2 | 3.41 | 4.843 | 0.053 |
| Raven progressive matrices | 42.9 | 7.56 | 27.7 | 4.59 | 0.251 | **2.436** |
| Intelligence quotient (education) | 102.0 | 10.88 | 100.3 | 4.17 | 3.631 | 0.205 |
| Intelligence quotient (age) | 109.0 | 13.54 | 104.0 | 5.72 | 3.249 | 0.481 |

*Student's t-test. Large effect sizes and significant ($p < 0.05$) differences are in bold characters.

0.5–30 Hz and sampled at 2,000 Hz. During the recording sessions, the attention of the subjects was directed to a magazine article that they were asked to read.

Responses were averaged separately for each stimulus type in each subject and the 0 $\mu$V baseline was determined as the mean amplitude of the pre-stimulus period. To quantify the MMN, the evoked response to the standard tone was subtracted from the corresponding deviant stimulus response and its amplitude (value relative to baseline) and latency at peak were measured over the midline electrodes, F3 and F4. Similarly, the P3a component of the ERP was obtained by subtracting the response to the standard tone from that to the novel stimuli and its amplitude (value relative to the baseline) and latency at peak were measured over the same five electrodes. Moreover, the amplitude (value relative to baseline) and latency at peak for the N100 component of the auditory evoked potential obtained with the standard stimuli were measured at Fz, Cz, Pz, F3 and F4.

### 2.4. Statistical analysis

In order to take into account the different education level of the two groups of subjects (see Table 1) due to generational reasons, the statistical analysis of the differences of the parameters of interest was carried out by means of the analysis of covariance (ANCOVA), using education level (years) as a covariate. When applied to two groups of subjects, as in this case, the ANCOVA provides results similar to those of a simple t-test with the added value of the correction for the covariate(s), which in this case was the education level.

However, this study was most probably underpowered because of the limited number of subjects available and, thus, we expected our results to be probably affected by type II errors. In order to deal with this, we also calculated the effect size using the Cohen's d value [23]. Cohen's d is defined as the difference between two means divided by the pooled standard deviation for those means. According to Cohen, 0.2 is indicative of a small effect, 0.5 of a medium and 0.8 of a large effect size. The commercially available software STATISTICA (data analysis software system), version 6, StatSoft Inc. (2001) was used for all statistical tests.

## 3. Results

Table 1 reports demographic and neuropsychological features of the two groups of subjects recruited for this study. Besides the obvious difference in age, in agreement with our recruitment criteria, the two groups clearly differed for education with the old subjects having attended school for only 5.7 years on average (10 years less than young subjects). This reflects the social-economic changes occurred in the recent history of the geographic area where the study was conducted. As this difference could have influenced the results of all measurements carried out in this study, we performed the subsequent statistical comparison between the two groups by means of the ANCOVA, with education used as a covariate, in order to correct for its effects on the results. For this reason, even if some large size effects were evident in the comparisons between the performance of the two groups at neuropsychological testing (Cohen's "d" $\geqslant 0.8$), none of the comparisons was found to indicate a significant difference.

Similarly, in Tables 2 and 3, all the comparisons between the ERP parameters obtained from the two groups failed to reach statistical significance, even if a difference could be observed in the group mean values in some instances, especially for N100 latency over F3, Cz and Pz at ISI 2,400 ms. Figure 1 shows, as examples, the ERP waveforms obtained from the two groups of subjects at ISI 2,400 ms.

Table 2
Comparison between ERP parameters obtained from the two groups of subjects at ISI 800 ms

| | Young adults ($n$ = 14) | | Old subjects ($n$ = 13) | | | |
|---|---|---|---|---|---|---|
| | Mean | S.D | Mean | S.D. | ANCOVA (F) | Cohen's "d" |
| N100 | | | | | | |
| Fz latency, ms | 117.8 | 3.93 | 118.6 | 5.46 | 0.101 | −0.166 |
| Fz amplitude, $\mu$V | −3.6 | 1.85 | −4.5 | 2.94 | 0.681 | 0.365 |
| Cz latency, ms | 116.4 | 3.40 | 118.3 | 5.18 | 0.003 | −0.428 |
| Cz amplitude, $\mu$V | −4.5 | 1.69 | −4.6 | 3.19 | 0.111 | 0.075 |
| Pz latency, ms | 115.7 | 3.17 | 119.3 | 6.71 | 0.593 | −0.697 |
| Pz amplitude, $\mu$V | −3.1 | 1.19 | −3.2 | 2.40 | 0.253 | 0.044 |
| F3 latency, ms | 116.4 | 3.70 | 118.8 | 6.67 | 0.149 | −0.441 |
| F3 amplitude, $\mu$V | −3.6 | 1.99 | −4.1 | 2.97 | 1.148 | 0.220 |
| F4 latency, ms | 118.1 | 4.51 | 119.7 | 7.14 | 0.003 | −0.259 |
| F4 amplitude, $\mu$V | −3.3 | 1.70 | −4.4 | 3.25 | 0.484 | 0.427 |
| MMN | | | | | | |
| Fz latency, ms | 180.5 | 33.84 | 170.5 | 23.06 | 0.051 | 0.347 |
| Fz amplitude, $\mu$V | −3.2 | 1.60 | −2.9 | 1.43 | 1.121 | −0.211 |
| Cz latency, ms | 176.3 | 35.35 | 171.5 | 23.31 | 0.653 | 0.161 |
| Cz amplitude, $\mu$V | −3.1 | 1.39 | −2.8 | 1.29 | 2.086 | −0.224 |
| Pz latency, ms | 191.2 | 27.42 | 172.2 | 24.34 | 0.031 | 0.734 |
| Pz amplitude, $\mu$V | −2.5 | 1.02 | −2.0 | 1.02 | 1.437 | −0.466 |
| F3 latency, ms | 182.9 | 26.69 | 169.5 | 23.04 | 0.057 | 0.537 |
| F3 amplitude, $\mu$V | −2.9 | 1.50 | −2.6 | 1.09 | 0.601 | −0.224 |
| F4 latency, ms | 185.0 | 29.81 | 167.8 | 22.71 | 0.053 | 0.650 |
| F4 amplitude, $\mu$V | −2.9 | 1.66 | −2.8 | 1.85 | 0.946 | −0.073 |
| P3a | | | | | | |
| Fz latency, ms | 352.4 | 34.23 | 357.8 | 30.93 | 0.026 | −0.166 |
| Fz amplitude, $\mu$V | 5.0 | 2.84 | 5.3 | 2.65 | 1.969 | −0.136 |
| Cz latency, ms | 351.1 | 33.29 | 357.2 | 31.31 | 0.004 | −0.188 |
| Cz amplitude, $\mu$V | 5.3 | 3.08 | 6.6 | 2.71 | 1.894 | −0.463 |
| Pz latency, ms | 350.5 | 31.58 | 361.0 | 33.73 | 0.164 | −0.320 |
| Pz amplitude, $\mu$V | 4.7 | 2.84 | 5.8 | 2.35 | 0.508 | −0.424 |
| F3 latency, ms | 349.4 | 35.68 | 355.9 | 29.88 | 0.016 | −0.196 |
| F3 amplitude, $\mu$V | 3.8 | 2.11 | 4.2 | 2.09 | 1.209 | −0.168 |
| F4 latency, ms | 350.6 | 34.45 | 357.1 | 32.20 | 0.009 | −0.195 |
| F4 amplitude, $\mu$V | 4.2 | 2.37 | 4.5 | 2.63 | 2.615 | −0.097 |

## 4. Discussion

Numerous neurodegenerative diseases lead to dementia; in many cases the diagnosis is difficult at a pre-clinical or sub-clinical stage, although this is the optimal time to introduce a rehabilitative therapy accompanied by medical interventions, in some circumstances [24]. Therefore it is of clinical relevance to measure complex higher order cognitive functions with techniques which are sensitive to subtle deficits [25]. This could also be the case of some sleep disorders causing mild cognitive deficits which are demonstrated to benefit from some treatments, such as modafinil for narcolepsy [26] and continuous positive airway pressure for obstructive sleep apnoea syndrome [27]. The N100, the MMN and the P3a components which are elicited in passive task conditions can be used also with patients who are not able to fully cooperate because of their motor or cognitive-behavioral impairment [6]. Moreover, paradigms having a good test-retest reliability and stability such as the P300, the MMN, the N100 can be used in follow-up studies (i.e. before and after medication) at the group level and, possibly, at the individual level too [28,29].

It is noteworthy that ERPs have some disadvantages such as: low spatial resolution, dependence on fluctuations of arousal [30], the not completely solved reference problem [31] and the need of averaging that makes assessment of ERP components critically dependent on latency jitter [32]. However, cognitive evoked potentials have many advantages: a fine temporal resolution, the method allows differential assessments of the patient's responsiveness to a large array of stimulus conditions, the equipment is relatively inexpensive, sometimes the results are unequivocal or may reflect cognitive decline more sensitively than neuropsychological tests in the longitudinal follow-up of patients [33,34].

Table 3
Comparison between ERP parameters obtained from the two groups of subjects at ISI 2,400 ms

| | Young adults ($n = 14$) | | Old subjects ($n = 13$) | | | |
|---|---|---|---|---|---|---|
| | Mean | S.D | Mean | S.D. | ANCOVA (F) | Cohen's "d" |
| N100 | | | | | | |
| Fz latency, ms | 116.8 | 6.71 | 119.3 | 6.16 | 0.004 | −0.397 |
| Fz amplitude, $\mu$V | −7.3 | 2.83 | −8.3 | 3.91 | 2.089 | 0.293 |
| Cz latency, ms | 116.0 | 4.89 | 119.6 | 6.00 | 0.079 | −0.667 |
| Cz amplitude, $\mu$V | −8.6 | 2.80 | −8.4 | 4.02 | 0.894 | −0.050 |
| Pz latency, ms | 115.1 | 4.37 | 120.2 | 6.33 | 0.966 | **−0.948** |
| Pz amplitude, $\mu$V | −5.5 | 2.05 | −5.4 | 3.14 | 0.352 | −0.037 |
| F3 latency, ms | 114.6 | 7.38 | 120.8 | 9.16 | 0.001 | −0.750 |
| F3 amplitude, $\mu$V | −7.0 | 2.74 | −7.7 | 4.09 | 1.419 | 0.200 |
| F4 latency, ms | 116.5 | 7.16 | 119.9 | 7.81 | 0.001 | −0.452 |
| F4 amplitude, $\mu$V | −6.3 | 2.67 | −7.9 | 4.26 | 1.267 | 0.442 |
| MMN | | | | | | |
| Fz latency, ms | 187.1 | 41.00 | 195.1 | 25.87 | 3.163 | −0.235 |
| Fz amplitude, $\mu$V | −3.3 | 2.31 | −2.7 | 1.40 | 0.107 | −0.309 |
| Cz latency, ms | 188.5 | 40.38 | 194.1 | 24.56 | 1.898 | −0.168 |
| Cz amplitude, $\mu$V | −3.0 | 1.73 | −2.2 | 2.13 | 0.293 | −0.415 |
| Pz latency, ms | 197.5 | 37.75 | 197.0 | 21.68 | 0.464 | 0.016 |
| Pz amplitude, $\mu$V | −2.1 | 0.95 | −2.0 | 1.40 | 0.125 | −0.046 |
| F3 latency, ms | 189.6 | 42.56 | 190.9 | 28.85 | 1.866 | −0.036 |
| F3 amplitude, $\mu$V | −2.9 | 1.53 | −2.8 | 1.36 | 0.299 | −0.072 |
| F4 latency, ms | 189.2 | 45.93 | 193.0 | 31.02 | 2.341 | −0.098 |
| F4 amplitude, $\mu$V | −3.1 | 2.20 | −2.2 | 1.20 | 0.254 | −0.472 |
| P3a | | | | | | |
| Fz latency, ms | 357.5 | 29.84 | 365.1 | 36.87 | 0.277 | −0.228 |
| Fz amplitude, $\mu$V | 3.8 | 3.01 | 6.0 | 4.00 | 1.141 | −0.602 |
| Cz latency, ms | 354.3 | 28.53 | 363.2 | 34.67 | 0.230 | −0.278 |
| Cz amplitude, $\mu$V | 5.0 | 2.66 | 7.2 | 4.32 | 0.654 | −0.612 |
| Pz latency, ms | 354.3 | 26.42 | 369.3 | 31.26 | 0.952 | −0.516 |
| Pz amplitude, $\mu$V | 5.2 | 1.68 | 7.0 | 3.89 | 0.004 | −0.589 |
| F3 latency, ms | 351.1 | 29.17 | 361.5 | 38.31 | 0.616 | −0.304 |
| F3 amplitude, $\mu$V | 3.4 | 1.87 | 5.2 | 3.55 | 0.365 | −0.659 |
| F4 latency, ms | 357.2 | 31.87 | 361.5 | 37.78 | 0.278 | −0.124 |
| F4 amplitude, $\mu$V | 2.9 | 2.38 | 4.9 | 3.00 | 0.349 | −0.744 |

Large effect size in bold characters.

That the human frontal lobes are particularly vulnerable to age-related deterioration has been frequently invoked as an explanation of functional decline in aging [35]. This "frontal aging hypothesis" could be addressed by an impairment of the N100, the MMN and the P3a; we demonstrated that pre-old subjects do not show this psychophysiological feature yet. In another study N100 and MMN were found to be less affected by age than other components such as N2b and P3b [36]. The stability of auditory discrimination and novelty processing in aging was found despite a lower level of education in comparison to young adults. The trend towards lengthening of the N100 latency over some electrodes found in this study seems to be caused by the lower education in the pre-old group. In the geographic area where the study was conducted, this disparity in education between young and old people is a social feature to be taken into account as clinical studies need to get inside real cultural conditions. However, the result of ERP stability with aging despite lower educational level reinforces that the components of the auditory information processing studied here are stable and therefore they could be successfully used to detect pre-clinical or sub-clinical cognitive changes presumably occurring in neurodegenerative disease and partly in sleep disorders.

MMN is ideal for addressing whether working memory impairment is due to premature trace decay, in research protocols, varying the ISI [9,10]. We have to admit that our ISI of 2,400 ms might be seen as a potential limitation of this study as we did not test ISI around 3,000 ms as previously reported in aging, schizophrenia, neurodegenerative dementias and Parkinson's disease (see [10–12] for review). However, we have also utilized novel stimuli that have rarely been used before in studies on auditory working memory with long ISIs.

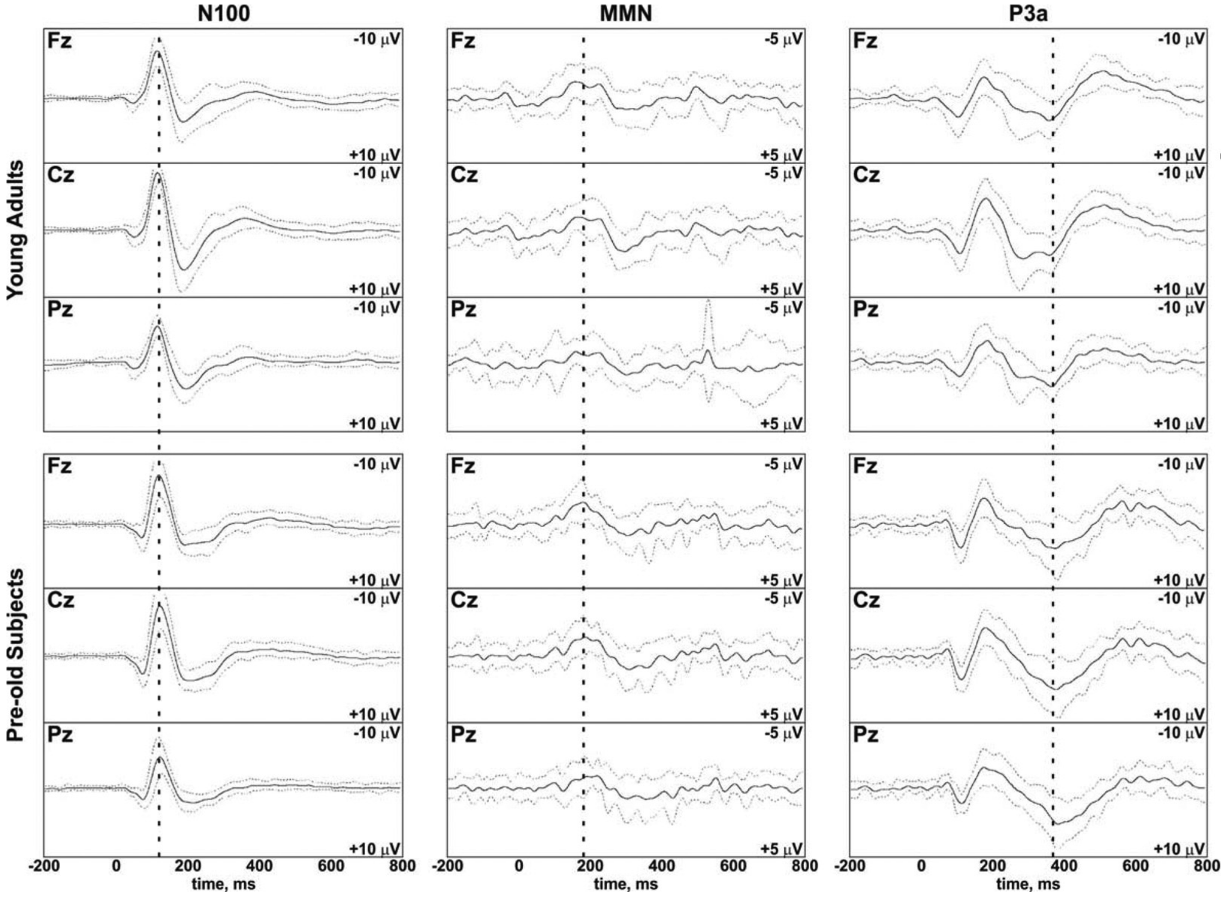

Fig. 1. ERP waveforms obtained from the two groups of subjects at ISI 2,400 ms. Only the midline derivations are shown. N100 was measured from the responses obtained after the standard stimuli (left panels); for the MMN, the evoked response to the standard tone was subtracted from the corresponding deviant stimulus (middle panels); finally, the P3a component was obtained by subtracting the response to the standard tone from that to the novel stimuli (right panels). The vertical dotted lines show the approximate peak time point of these components.

The addition of another ISI condition to our protocol would have caused an unacceptable lengthening of the recording sessions.

It has been demonstrated that a decrease of phase-locking and phase coherence caused by aging was accompanied by increased activity in the late (P3, 280–450 ms) theta response over the frontal and temporo-frontal areas, and that this late component may reflect the effect of higher-order cognitive functions [37]. Increased activity in the elderly – in our study we have found stability – it is considered to be associated with enhanced memory performance [38]. Possibly, for an older person who needs to perform a task as efficiently as a young one it is necessary to increase the activity in the bilateral frontal and temporo-frontal areas by increasing the late theta component. The compensatory model hypothesis, which has already been proposed in the literature [39,40] suggests that compensatory activation can be considered as a feature of normal aging [41]. This is in part in line with our findings that demonstrate a stability of auditory information processing in healthy and not too aged subjects in whom compensatory mechanisms may occur. However the number of recording electrodes was relatively small and the our analysis concerned only amplitude and latency at peak; thus, a structural analysis of brain connectivity was not feasible and this represents a limitation of our analysis.

Our results are also in line with those concerning preattentive components, including N100, demonstrating that physiological aging does not affect auditory gating in amplitude measures. Inhibition of redundant information seems to be preserved with normal aging. Therefore, further studies are warranted to evaluate sensory gating as a suitable biomarker of underlying neurodegenerative disease [5].

In conclusion, we can affirm that if strictly normal subjects are recruited, as in this study, the pre-old age period is characterized by a stability of the auditory discrimination and novelty processing. This stability is important for the arrangement of normative data that might prove to be sensitive for the detection of subtle and, possibly, preclinical changes due to abnormal or pathological brain aging, involving especially the frontal lobes. Thus, we suggest that a research agenda might be planned involving larger numbers of normal subjects, with age divided by decades, in whom not only ERPs are recorded but also neuroimaging techniques can be paralleled.

## Acknowledgments

The authors acknowledge the valuable contribution of Annalisa Galeano in data acquisition and that of all the volunteer subjects who joined the test. This study was supported by the Italian Ministry of Health ("Ricerca Corrente" and "Cinque per Mille").
